# Tools and Methods for Diagnosing Developmental Dysgraphia in the Digital Age: A State of the Art

**DOI:** 10.3390/children10121925

**Published:** 2023-12-14

**Authors:** Jérémy Danna, Frédéric Puyjarinet, Caroline Jolly

**Affiliations:** 1 University of Toulouse, Centre National de la Recherche Scientifique (CNRS), Laboratoire Cognition, Langues, Langages, Ergonomie (CLLE), 31058 Toulouse, cedex 9, France; jeremy.danna@cnrs.fr; 2 UFR de Médecine de Montpellier-Nîmes, Institut de Formation en Psychomotricité de Montpellier, 2 rue Ecole de Médecine, CS 59001, 34060 Montpellier, cedex 2, France; frederic.puyjarinet@umontpellier.fr; 3 University Grenoble Alpes, University Savoie Mont-Blanc, Centre National de la Recherche Scientifique (CNRS), Laboratoire de Psychologie et NeuroCognition (LPNC), 38000 Grenoble, France

**Keywords:** handwriting, developmental dysgraphia, product, process, diagnosis

## Abstract

Handwriting is a complex perceptual motor task that requires years of training and practice before complete mastery. Its acquisition is crucial, since handwriting is the basis, together with reading, of the acquisition of higher-level skills such as spelling, grammar, syntax, and text composition. Despite the correct learning and practice of handwriting, some children never master this skill to a sufficient level. These handwriting deficits, referred to as developmental dysgraphia, can seriously impact the acquisition of other skills and thus the academic success of the child if they are not diagnosed and handled early. In this review, we present a non-exhaustive listing of the tools that are the most reported in the literature for the analysis of handwriting and the diagnosis of dysgraphia. A variety of tools focusing on either the final handwriting product or the handwriting process are described here. On one hand, paper-and-pen tools are widely used throughout the world to assess handwriting quality and/or speed, but no universal gold-standard diagnostic test exists. On the other hand, several very promising computerized tools for the diagnosis of dysgraphia have been developed in the last decade, but some improvements are required before they can be available to clinicians. Based on these observations, we will discuss the pros and cons of the existing tools and the perspectives related to the development of a universal, standardized test of dysgraphia combining both paper-and-pen and computerized approaches and including different graphomotor and writing tasks.

## 1. Handwriting: Acquisition and Role

Handwriting, considered language by hand, is a complex perceptual motor task involving attentional, perceptual, linguistic, and fine motor skills. It occupies a large proportion of children’s daily activities at school [1,2] and is the basis, together with reading, of the acquisition of higher-level skills such as spelling, grammar, syntax, and text composition. A relationship between the mastery of handwriting movement and the quality of writing content has been established both at the semantic level in text production [3] and at the orthographic level in word formation [4]. If children pay too much attention to handwriting movements, they may have difficulties in the allocation of cognitive resources to higher-level processes.

From a developmental perspective, handwriting originates from drawing, from which it slowly differentiates as the child grows. In younger children, the quality of drawings is correlated to the quality of handwriting [5]. Then, with the acquisition of handwriting, this relationship between drawing quality and writing quality remains correlated but attenuates, with more discrepancies [6]. The formal acquisition of handwriting begins around the age of 5 at preschool, and its mastering requires about 10 years of practice and training. The automation of handwriting is partial at the age of 10 (5th grade) and is considered almost complete around the age of 14 (9th grade) (for a review, see [7]). During acquisition, handwriting evolves first in terms of quality (primarily between 1st and 5th grade), then in terms of speed (from 4th grade onward, essentially). Efficient, fully automated handwriting relies on a balance of speed and quality; it should be fast enough to allow the retranscription of a course or the transcription of ideas and of sufficient quality to be readable by the writer and by others.

## 2. Handwriting Deficits

Despite the correct learning and practice of handwriting, some children never master this skill to a sufficient level of automation (reviewed in [8,9,10]). These handwriting deficits, referred to as developmental dysgraphia in children, have been defined as a written-language disorder that concerns mechanical writing skills in children of average intelligence and with no distinct neurological or perceptual motor deficits [11]. Currently, dysgraphia is not recognized as a disorder per se in the Diagnostic and Statistical Manual of Mental Disorders, fifth edition (DSM-5) [12], or the International Classification of Diseases, 11th edition (ICD-11). The DSM-5 only mentions “deficits in the fine motricity required for handwriting” in the chapter dedicated to the development and evolution of learning disorders. Due to the diversity of methodological approaches and the absence of a consensual definition, the exact prevalence of dysgraphia is not known and probably differs between countries and writing systems.

Dysgraphia is generally found in association with neurodevelopmental disorders, namely dyslexia (DL), Developmental Coordination Disorder (DCD) and Attention Deficit Disorder/Hyperactivity Disorder (ADHD) [13,14,15,16,17,18]. Dysgraphia preferentially affects boys (3:1 ratio), most likely because of the prevalence of the associated disorders in boys [8,19]. Many studies have shown differences in handwriting deficits depending on the associated disorder [20,21,22,23,24,25,26]. DCD primarily affects handwriting quality [22,27,28] while DL affects both speed and, to a lesser extent, handwriting quality [26,29]. Children with comorbid DL and DCD present nearly the same profile of difficulties as children with DL, although with a much higher within-group variability. Comorbidity seems to lead to the addition of DCD and DL writing difficulties but without aggravation of the deficits in each of the two dimensions [24].

Dysgraphia can vary according to graphic and linguistic systems. Firstly, the perceptual and motor complexities of different graphic systems vary widely. Some graphic systems require many hours of practice to reach a comparable level of automation, while others are much easier to learn. For example, the Kanji system, which requires a minimum knowledge of 2136 essential kanji (*jōyō kanji*), according to the Japanese Ministry of Education, even though they are made up of a large number of strokes (up to 23 strokes for the most complex kanji), is much more complex than the Latin alphabet, which is based on 26 letters only. As a result, the risk of difficulty is much greater in the former than in the latter. Secondly, within the same graphic system, some linguistic systems are also more complex than others: in the grapheme–phoneme relationship, for example. Italian and English are examples of transparent and non-transparent languages, respectively, for which the amount of reading and writing practice can vary to reach the same level of expertise. Knowing the interaction between orthographic and graphomotor constraints [4], one may assume that the risk factor for developing dysgraphia is higher in the case of English than in the case of Italian, especially when dysgraphia is subsequent to dyslexia [30].

Given the central role of handwriting in the acquisition of other skills, these deficits can seriously hamper the acquisition of other skills [31,32,33]. It has been shown that, given equal content, the worst quotes are attributed to less legible school works [34], resulting in a decrease in the child’s self-esteem. Dysgraphia may thus impact the academic success of a child if it is not diagnosed and handled early [35,36]. To this end, different tools are available to allow researchers and clinicians to analyze the two dimensions of handwriting: the final product and the dynamic process that generates the trace [37,38].

Evaluation of the handwriting product refers to the static, spatial features of the written trace. This kind of analysis is performed afterward. This is the principle of many tests used in different countries (for a review, see [8]). The quality of the trace is evaluated based on different features such as letter size and form, the spatial organization of handwriting on the paper sheet, margins, etc. 

Evaluating the handwriting process refers to the analysis of the dynamic, kinematic, and temporal features of handwriting. Several types of variables can be analyzed, depending on the tools used for the evaluation: posture, finger and arm movements, pen grip and finger pressure on the pen, in-air and on-paper durations, pen velocity, pen pressure, etc. The increasing number of publications on the analysis of the handwriting process over the past years attests to the growing interest of researchers in this field (e.g., [39,40,41,42,43]).

The objective of this review is to make a concise listing of the tools and methods that are the most reported in the literature for the analysis of handwriting and the diagnosis of dysgraphia. Tools focusing on both the final handwriting product and the handwriting process will be considered. We will then discuss the pros and cons of the existing tools and the perspectives for the development of future tools.

## 3. Handwriting Tools Based on the Product

In order to list the diagnosis tools based on the analysis of the handwriting product, we searched two scientific browsers, PubMed and Google Scholar, using the following keywords: Handwriting, Assessment, Test, Tool, Quality, Evaluation, Battery, Children, Students, and Questionnaire.

The tools meeting our search criteria are listed in Table 1. We included only tools for which the following data were available: norms or age class, type of task, subdomain analyzed, and criteria evaluated.

Although mainly designed for a developmental population (from the age of 5 onward), some diagnosis tools can also be used on adults up to the age of 80 (QNST-3; [58]). The test duration is variable, from a few minutes to up to 30 min. This parameter is interesting because deficits may not be visible during the first few minutes of handwriting but may appear during a continuous handwriting task, as is the case in the classroom. The tasks used in the tests are of three main types: copying a text or a sentence, writing under dictation (letters, digits, words, or text), and spontaneous writing. These complementary tasks explore different aspects of handwriting. The copy task is the easiest and can be used with beginner writers. Moreover, it resembles the condition of the classroom, where children are often asked to copy texts. However, the reading component can pose problems for children with dyslexia, introducing a possible bias in the interpretation of the test results. The dictation task is ecological too, without the reading component, but the spelling processes and the orthographic components may again pose problems for children with dyslexia. Finally, the spontaneous writing task is likely to be the most relevant. The difficulty here is the establishment of norms, since the texts produced are all unique. The general criteria of legibility and quality are thus used in this case, which may provide a less fine-grained analysis of handwriting.

It should be noted that one test includes an analysis of texts produced at school: the TOLH (Test of Legible Handwriting [60]). Two others include writing from memory: the ETCH-M (Evaluation Tool of Children’s Handwriting—Manuscript [51]) and the MMHAP (Mac Master Handwriting Assessment Protocol [55]). Two tests also add another level of analysis thanks to two conditions in the copy task: normal speed and maximum speed (the DASH [49]). This approach is particularly interesting, since it mimics certain classroom conditions, and it is well-known that adding constraints (temporal or spatial) during handwriting helps reveal handwriting deficits [62,63]. Combining different tasks and/or conditions can provide a fine and detailed analysis of handwriting. It is worth noting that although these tasks are complementary, only three tests involve all three types: the BVSCO-3 [46], the ETCH-M [51], and the MMHAP [55]. 

The majority of the tests listed in Table 1 analyze handwriting quality using different criteria such as legibility, letter form, the spatial organization of letters or words, alignment, etc. Some tests also measure handwriting speed by evaluating the number of characters or letters (BHK [44]; French adaptation [64]; BHK-ado [45]; BVSCO-3 [46]; CHES-M [48]; ETCH-M [51]; EVEDP [52]; MMHAP [55]; MHA [56,57]) or the number of words produced in a fixed period of time (DASH [49]; EVEDP [52]). Since a universal, gold-standard test for the diagnosis of dysgraphia is not available, it is sometimes necessary to combine several tests to perform an optimal clinical assessment. The DASH test appears to be the most complete one, since it includes various types of tasks and different constraints of writing and it requires about 15 min of writing. Its weakness is that it only evaluates handwriting speed.

Finally, we should also mention the existence of questionnaires, which can be interesting to use to complement the other tests. Indeed, these questionnaires provide subjective information about the evaluation of handwriting quality by the teacher or the child, which can be useful in the perspective of a rehabilitation program. In addition, these questionnaires could also be used for the screening of children with handwriting difficulties on a larger scale. The Handwriting Proficiency Questionnaire (HPSQ [65]) has been developed in different languages for children from 7 to 14 years old. It has to be completed by adults (teachers or clinicians). An adaptation of this questionnaire, the HPSQ-C, was developed later to inform about a child’s perception of his/her handwriting quality. This autoquestionnaire has been shown to be suitable for the identification of handwriting deficiency among school-aged children and to be appropriated for clinical use [66]. Likewise, the “questionnaire for children” [67] is an autoquestionnaire in which children self-report their handwriting quality and difficulties. It targets children from 1st to 5th grade, but only a French version is available. 

Another important point to consider when choosing which test to use is the existence of standards. Table 2 presents the psychometric properties of the main tests used both in research and in clinical practice. A number of tests have relatively good inter-rater and test–retest reliabilities (the French adaptation of the BHK, for example [64]), while others have reached high validity-related standards (the MHA [56] and the TOLH [60], for example).

More recently, a few computerized diagnostic tools based on the analysis of the final products of handwriting have also been developed. They are listed in Table 3. 

These algorithms are all based on pattern recognition methods using images of letters, digits, words, or sentences. They use a large database of images from which the characteristic features of “poor writing” are extracted and analyzed using machine learning approaches. The performances of computer tools are evaluated using a series of criteria. Precision, also called the positive predictive value, is defined as the number of correct classifications of dysgraphic children divided by the total number of classifications. Sensitivity represents the true-positive detection rate (the correct classification of children with dysgraphia). Specificity represents the true-negative detection rate (the correct classification of typically developing children). 

As shown in Table 3, the performance of these classification tools is below that of the paper-and-pen tools listed above (73% for [71]; 79.7% for [72]). The only exception is TestGraphia, the algorithm developed by Dimauro et al. [70], with good performances very close to that of the original BHK test. It analyzes the same criteria as the original BHK test [68] but using scanned images of the BHK texts. The sensitivity of TestGraphia is 83%, and its specificity is 98%. This algorithm thus seems very promising for the future development of computerized diagnostic tools.

## 4. Handwriting Tools Based on the Process

Collecting the spatio–temporal characteristics of a written trace has become possible thanks to the development of digital tablets. The principle is simple: the tablet records the x, y, and sometimes z (up to 2 cm) positions of the pen with a high frequency (every 5 or 10 milliseconds), as well as the time, the pen pressure, and the angle of the pen to the tablet. From these data, a large variety of static (size, alignment…), kinematic (speed, acceleration, jerk…), and dynamic (pen pressure, pen tilt…) features can be calculated. To avoid the undesirable effects of loss of surface roughness (e.g., [1]), a sheet of paper must be attached to the digital tablet and an ink pen compatible with the tablet must be used.

Over the last decades, a growing number of studies have focused on the development of tools for the diagnosis of dysgraphia using digital tablets. In this review, we present a non-exhaustive overview of these tools, which are not yet available to clinicians (Table 4).

The different digital tools for the diagnosis of dysgraphia, presented in Table 4, combine dynamic, kinematic, and static features extracted from handwritten tracks. These features are then analyzed using mainly machine learning approaches to classify the data (i.e., classifiers). These tools differ by the natures of the tasks analyzed (handwriting or graphomotor tasks), the sizes of the datasets, and the computational approaches used to analyze the data.

Of the 22 studies reported here, four used graphomotor tasks; the others used handwriting alone or a combination of handwriting and drawings. It is interesting to mention that several studies have used tasks that have been validated in clinical practice, such as the BHK [39,73,76,82], the BVSCO2 [78], or the Minnesota Handwriting Assessment (MHA [80]).

The size of the dataset used varied between 35 and 580 participants, and the children included in the different studies were between 5 and 15 years of age.

Nine studies used classical statistical comparisons to identify discriminative features between groups (in blue in Table 4; [39,77,78,80,81,82,83,87,91]). The others (in black in Table 4) used different algorithms of machine learning (Random Forest, Support Vector Machine, Convolutional Neuron Network, etc.) to classify children into different groups. These methods are called “supervised learning approaches”, since the algorithm was trained to identify groups that were previously labeled. Most of the studies reported here present a simplistic classification of children in two groups: with or without dysgraphia. Only one study classified the children into four groups: typically developing, with mild dysgraphia, with mean dysgraphia, and with severe dysgraphia [89]. This approach is interesting, since it considers dysgraphia as a continuum of severity. This is probably closer to reality than a dichotomic classification, as has been recently suggested by Lopez and Vaivre-Douret [92], who have described three levels of handwriting disorders in children from 1st to 5th grade: mild disorder, moderate disorder, and dysgraphia.

The tools based on the analysis of handwriting samples obtained the best classification performance. For example, Asselborn et al. [73] reached a sensitivity of 96.6% and a specificity of 99%, and Mekyska et al. [86] reached a sensitivity of 96%. It is worth noting, however, that the excellent performances obtained in [73] must be considered with caution, since they may be biased by the fact that the authors only included participants with severe dysgraphia [93]. The most discriminative features between children with and without dysgraphia varied among the studies but generally included a larger size in dysgraphic handwriting, numerous velocity variations, a lower mean speed, increased lift and stop duration, and variations in the pen angle to the tablet.

The tools based on the analysis of drawing samples appeared promising too, although their performances were slightly lower than of those based on handwriting. For instance, the algorithm developed by Mekyska et al. [87] obtained a sensitivity of 90%. The idea that dysgraphia can be identified based on graphomotor tasks suggests that it can be independent from higher-order processes, namely linguistic ones. Developing diagnostic tools based on drawings is interesting for two reasons: these tools would be more universal, since they are independent of the language and the alphabet, and they can be used with younger children to identify “at-risk” children, which could be handled earlier.

Developing a computer tool for the diagnosis of dysgraphia is not trivial, as attested to by the variability in the performances of the tools presented in Table 4. Several reasons can explain these differences. First, the variety of the tasks used and the number of participants led to large differences in the sizes of the databases, which was a critical determinant in a classifier’s performances. Second, a large panel of machine learning approaches was used, with different numbers of features analyzed among studies. Although certain classification methods appeared better than others (Random Forest, for example), none have currently reached excellent performances. Since the interest of researchers in these tools is growing, it seems obvious that their efficiency will rapidly be improved. To do so, however, a number of key elements will be important to consider. First, it will require the constitution of large databases of handwriting and drawing samples from children that are perfectly characterized from a clinical point of view. It will also be necessary to estimate the severity of dysgraphia and not only provide a dichotomic classification of children with or without dysgraphia, as proposed by Sihwi et al. [89]. Moreover, other processes involved in handwriting, such as visuomotor aspects, which are currently being investigated [94], would be interesting to include in future diagnostic tools. Finally, it is also worth noting that diagnostic tools fully integrated into the pen and using machine learning approaches are also under investigation [95,96,97]. 

## 5. Perspectives: Toward a Universal Standardized Test of Dysgraphia? 

Since dysgraphia is a very heterogenous disorder encompassing a large variety of difficulties, it would be interesting to think about developing a reliable, comprehensive, and universal diagnostic tool for dysgraphia, combining computer and paper-and-pen tools. The main diagnostic features assessed with the paper-and-pen tools can be complemented with the computerized tools, which would provide precise information about the specific handwriting difficulties of each child (for a review, see [43]).

**Figure 1:**
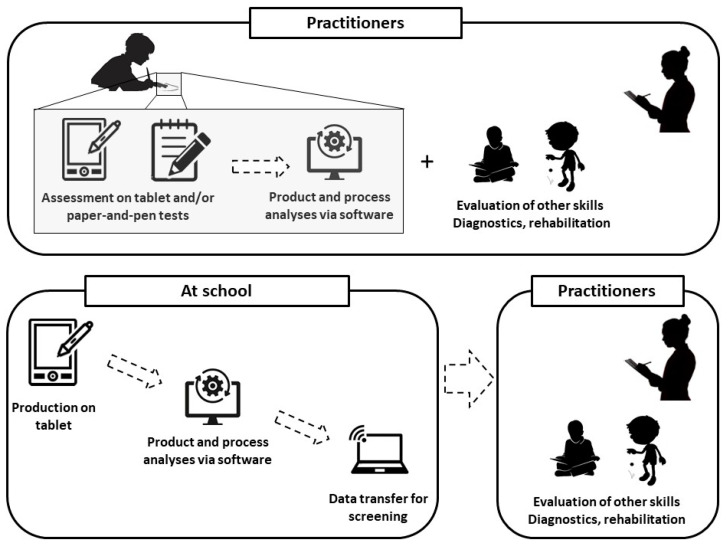
Examples of the complementary use of paper-and-pen tests and computerized tools for the diagnosis and rehabilitation of handwriting deficits. In addition to handwriting traces collected by clinicians using standard paper-and-pen tools, written samples could also be collected using tablets and/or smart pens, both by clinicians (top panel) and by teachers in their classrooms (bottom panel). Kinematic features reflecting the handwriting process could then be extracted from these computerized traces and transmitted by the teachers to the clinicians. The practitioner could combine all these static, dynamic, and kinematic parameters with the evaluation of other skills to eventually propose a complete diagnosis and an adapted rehabilitation program for the children.

Several important points need to be considered for the development of such an instrument. First, an “ideal” diagnostic tool would probably combine computer and paper-and-pen approaches, since they are complementary and provide distinct information on the writing process and product, respectively. A fully computerized tool could also be envisaged, provided that it is complemented by the assessment of a clinician, who must remain the reference assessor. Indeed, the spread of tablets and the rapidity of computerized analyses could allow the collection of written samples in school or at children’s houses; they could then be sent to a clinician. Standard pen-and-paper tools could subsequently be used in case the computer tools detect a risk of dysgraphia in the child’s handwritten productions in order to firmly confirm the diagnosis. From this perspective, the goal of the computer tools is thus not to replace the clinician or the existing, validated tests but to help in screening larger populations of children and in facilitating clinician diagnosis (Figure 1). In addition, these tools could provide valuable information on the process of handwriting itself by identifying dynamic or kinematic features that may be altered in each particular child. This information would be very relevant to clinicians, since it would offer cues for an individualized rehabilitation of handwriting.

Second, using a combination of tasks targeting different skills seems crucial to providing more information about handwriting difficulties. Indeed, some children with dysgraphia may succeed at certain tasks and thus be undiagnosed if only a single one is used. Combining different tasks in a unique test would thus greatly increase its efficacy, as has been previously suggested by Safarova et al. [98]. Namely, the test should include spontaneous handwriting, the copying of words and/or sentences, writing to dictation, digit writing, writing under speed and accuracy constraints, and drawing and/or graphomotor tasks. Temporal (i.e., speed) and spatial (i.e., size) constraints add a cognitive load and are known to increase handwriting difficulties [16,61,62]. With regard to the spontaneous production task, we could, for example, ask the participant to write a seven-sentence text corresponding to the writer’s ideal weekly schedule. This would enable a specific analysis of the days of the week to be made, which would be common to all texts produced. As mentioned above, the addition of graphomotor and/or drawing tasks, which are language-independent, would enable the targeting of younger children more than the existing tests and thus the earlier detection and handling of children “at-risk” of dysgraphia. In addition, it would provide a universal test, allowing comparisons between countries and alphabetical systems. In addition, the test needs to last at least 20 min in order to enhance the difficulty of the task and induce fatigue. Finally, completing the test with a self-questionnaire would enable the clinician to better characterize the difficulties experienced by the writer.

Thirdly, the choice of the cohort of participants would be crucial. A large developmental window ranging from 5 to at least 15 years old should be included, and the content of the test should be adapted depending on the age and/or class of the child and the level of handwriting automation. The number of participants should be important enough to allow machine learning approaches. It would also be important to include children presenting dysgraphia in various clinical contexts and precisely characterized from a clinical perspective. This would enable the evaluation of the severity of dysgraphia, which could eventually be an additional evaluation criterion provided by the diagnostic tool. Finally, participants should be recruited in multiple sites that are representative of different socio-economic and educational statuses.

Developing such a complete diagnostic tool implies the collection of large databases of handwriting and drawing samples in different places around the world. This would be possible with the implication of a consortium of laboratories and clinicians. Besides the diagnostic tool itself, the benefits of these developments would be twofold: (i) from a clinical perspective, it would allow the estimation of the prevalence of dysgraphia in different countries, and it would further tailor rehabilitation programs to the characteristics of handwriting difficulties; and (ii) from a research perspective, it would provide large, annotated databases that could be freely available to researchers working in the fields of graphonomics, whether in educational, clinical, or human movement sciences.

## Figures and Tables

**Table 1:** List of the most commonly used tools for the diagnosis of dysgraphia in children based on the analysis of the handwriting product (presented in alphabetical order). N.A.: not available.

Tool Name	Reference	Age/Class	Duration of Test	Language	Task(s)	Subdomains
BHK: Brave Handwriting Kinder	[44]	1st to 5th grade	5 mn	Multi-language	Copy	Quality Speed
BHK Ado: Rapid Writing Evaluation Scale for Adolescents (*Echelle d’Evaluation Rapide de l’Ecriture Chez l’Adolescent*)	[45]	6th to 9th grade	5 mn	French	Copy	Quality Speed
BVSCO-3: Test for the Evaluation of Writing and Orthographic Ability, 3rd ed.	[46]	6–14 y	Variable	Multi-language	Copy Dictation Spontaneous production	Speed % of errors
CHES: Children’s Handwriting Evaluation Scale	[47]	3rd to 8th grade	2 mn	English	Copy	Quality Fluency
CHES-M: Children’s Handwriting Evaluation Scale—Manuscript Writing	[48]	1st to 2nd grade	2 mn	English	Copy	Quality Fluency
DASH: Detailed Assessment of Speed of Handwriting	[49]	9–16 y	14 mn	English	Alphabet copy at normal and high speed Spontaneous production	Speed
DRHP: Diagnosis and Remediation of Handwriting Problems	[50]	From 3rd grade	Variable	English	Spontaneous production from images observation	Quality
ETCH-M: Evaluation Tool of Children’s Handwriting—Manuscript	[51]	1st to 2nd grade	15–20 mn	English	Copy Dictation Spontaneous production Handwriting from memory	Quality Speed
EVEDP: Evaluation de la Vitesse d’Ecriture—Dictée Progressive	[52]	2nd to 5th grade	Variable	French	Dictation	Speed
HHE: Hebrew Handwriting Evaluation	[53]	6–18 y	5 min	Hebrew	Alphabet Copy Dictation Spontaneous production	Quality Speed
HLS: Handwriting Legibility Scale	[54]	9–14 y	10 mn	English	Spontaneous production	Quality
MMHAP: Mac Master Handwriting Assessment Protocol	[55]	Preschool to 6th grade	Variable	English	Copy Dictation Spontaneous production Handwriting from memory	Quality Speed
MHA: Minnesota Handwriting Assessment	[56,57]	1st to 2nd grade	2.5 mn	English	Alphabet Copy	Quality Speed
QNST-3 Revised: Quick Neurological Screening Test, 3rd ed. Revised	[58]	5–80 y	30 mn	English	Copy	Quality
SCRIPT: Scale of Children’s Readiness in Printing	[59]	N.A.	3–8 mn	English	Copy	Quality
TOLH: Test of Legible Handwriting	[60]	2nd to 12th grade	Variable	English	Spontaneous production Text composition at school	Quality
THS-R	[61]	6–18 y	N.A.	English	Alphabet Copy	Quality

**Table 2:** Psychometric properties of the main diagnosis tools for handwriting assessment. BHK: Brave Handwriting Kinder; CHES-M: Children’s Handwriting Evaluation Scale for Manuscript writing; DASH: Detailed Assessment of Speed of Handwriting; DRHP: Diagnosis and Remediation of Handwriting Problems; ETCH-M: Evaluation Tool for Children’s Handwriting—Manuscript; HHE: Hebrew Handwriting Evaluation; HPSQ: Handwriting Proficiency Screening Questionnaire; THS-R: Test of Handwriting; TOLH: Test of Legible Handwriting; N.A.: not available.

Test Name [Ref]	Number of Participants	Country of Validation	Validity	Inter-Rater Reliability	Test–Retest Reliability	Internal Consistency
BHK [11,44]	121	Netherlands	Content and construct	.71 to .89	.74 to .86	N.A.
BHK—French Adaptation [64]	837	France	Content and construct	.68 to .90	.80 to .92	N.A.
BHK Ado [45]	471	France	Construct	.24 to .66	N.A.	N.A.
BHK—Italian adaptation [68,69]	562	Italy	Content and construct	.82 to .93 for speed .42 to .63 for quality	N.A.	N.A.
CHES-M [48]	643	USA	N.A.	.85 to .93	N.A.	N.A.
DASH [49]	1163	Netherlands	Content and construct	.85 to .99	.50 to .92 .81	.88 to .94
DRHP [50]	300	UK	Construct	.61 to .65	N.A.	N.A.
ETCH-M [51]	N.A.	N.A.	N.A.	.75 to .92	.63 to .77	N.A.
HHE [53]	N.A.	Israël	Content and construct	.75 to .79	N.A.	N.A.
HPSQ [65]	230	Israël	Content and construct	.92	.84	.90
MHA [56,57]	N.A.	USA	Content and construct	.87 to .98	.58 to .94	N.A.
THS-R [61]	N.A.	USA	Construct	N.A.	.82	N.A.
TOLH [60]	1723	USA	Content and construct	.95	.90	.86

**Table 3:** List of the computerized diagnosis tools in children based on the analysis of the handwriting product. CNN: Convolutional Neural Network; RF: Random Forest; SVM: Support Vector Machine; ANN: Artificial Neural Network.

Ref	Age/Class	Characteristics of Participants	Task(s)	Language	Approach	Performances
[70]	7–10 y	Dysgraphic	BHK (5 lines)	Italian	Algorithms for document analysis	Sensitivity: 83% Specificity: 98% Precision: 96%
[71]	7–12 y	Dyslexic	Letter and digit writing	Malaysian	Machine learning (ANN)	Sensitivity: 73%
[72]	8–15 y	Typically developing and dysgraphic	Letters, syllables, words, pseudowords, and sentences	Slovak	Machine learning (CNN, RF, SVM, AdaBoost)	Precision: 79.7%

**Table 4:** List of the algorithms and computer tools for the diagnosis of handwriting deficits. In blue: tools based on statistical approaches; in black: tools based on machine learning approaches. ADHD: Attention Deficit/Hyperactivity Disorder; BEM: Beta-Elliptic Model; BVSCO-2—Test for the Evaluation of Writing and Orthographic Ability, 2nd ed.; CNN: Convolutional Neural Network; DG: dysgraphic children; FDM: Fourier Descriptor Model; FOD: Fractional Order Derivative; KNN: K-nearest Neighbors; MHA: Minnesota Handwriting Assessment; MLP: Mumti Layers Perceptron; P: precision; RBF: Radial Basis Function; RF: Random Forest; SE: sensitivity; SP: specificity; TD: typically developing children.

Reference	Age/Class	n	Tasks	Language/Alphabet	Approach	Criteria Analyzed	Performances
[73]	6–10 y	242 TD 56 DG	Copy of a text (BHK)	French	RF	Static Kinematic Pressure Pen tilt	SE: 96.6% SP: 99.2% P: 97.98%
[39]	5–12 y	390 TD 58 DG	Letters, words, sentences	French	PCA + K-means clustering	Static Kinematic	SE: 91% SP: 90%
[74]	10–13 y	39 TD 39 DG	Letters, words, sentences	Slovak	SVM	Kinematic	SE: 75.5%
[75]	7–11 y	262 TD 63 DG	Copy of graphic shapes	N.A.	SVM, RF, MLP, extra trees, AdaBoost, Gaussian Naive Bayes	Kinematic	SE: 75.1% (RF) SP: 72.1% (MLP) P: 73% (extra trees), 73.4% (RF)
[76]	7–11 y	458 TD 122 DG	Copy of a text (BHK)	French	SVM	Kinematic Spatial	SE: 91% SP: 81% P: 86%
[40]	8–15 y	63 TD 57 DG	Letters, syllables, words, pseudowords, sentences with speed constraints	Slovak	AdaBoost, RF, SVM	Kinematic	SE: 79.7% SP: 76.7% P: 80%
[77]	5–8 y	76 TD 28 DG	Copy of words (8y), graphic shapes (5 and 8 y)	Italian	Statistical comparisons between groups	Kinematic Pressure	N.A.
[78]	7–8 y	52 TD	Subtest of the BVSCO-2 (digits, sequences of small and large loops, words)	Italian	Statistical comparisons	Kinematic	N.A.
[79]	5 y	241 “at-risk of DG”	Copy of graphic shapes	N.A.	One-dimensional CNN	Kinematic	SE: 75% SP: 77% P: 76%
[80]	6–7 y	26 TD 9 DG	MHA	English	Statistical comparisons	Static Kinematic	N.A.
[81]	8–12 y	26 TD	Copy of graphic shapes	Czech	Q factor wavelet transform + statistical comparisons	Static Kinematic	P: 84%
		27 DG					
[82]	7–10 y	218 TD 62 DG	Copy of a text (BHK)	French	Statistical comparisons between groups (linear regression), clustering	Static Kinematic Pressure Pen tilt	N.A.
[83]	6–11 y	5 TD 9 ADHD	Dictation of letters and digits MHA	English	Statistical correlations between manual and digital data	Static Kinematic	N.A.
[84]	7–12 y	60	Copy of words, sentences, and graphic shapes	Latin	RF, decision tree, SVM	Kinematic	SE: 92.8% P: 92.6%
[85]	8–15 y	63 TD 57 DG	Letters, words, sentences	Slovak	KNN, SVM, RF, AdaBoost	Kinematic (on-surface and in-air)	SE: 78.5% P: 80.8%
[86]	8 y	27 TD	Letters	Hebrew	RF, linear discriminant analysis	Kinematic	SE: 96%
		27 DG					
[87]	8–9 y	61 TD 15 DG	Copy of patterns and figures	Czech	XG-Boost	Kinematic	SE: 90%
[88]	8–9 y	14 TD 14 DG	Copy of a text	Hebrew	Statistical comparisons between groups	Static Kinematic	N.A.
[42]	8–9 y	50 TD 49 DG	Copy of letters and sentences	Hebrew	SVM	Static Kinematic	SE: 90% SP: 90% P : 89.9%
[89]	8–11 y	32 TD	Spontaneous writing (sentences), drawings	Indonesian	SVM and RBF Kernel	Kinematic	P: 82.5%
[90]	8–9 y	33 TD 32 DG	Copy of a text	Czech	Tunable Q-factor wavelet transform, RF and SVM classifiers	Kinematic	SE: 88.7% SP: 83% P: 84.7%
[91]	8–9 y	30 TD 25 DG	Spontaneous writing of letters	Czech	Correlation between the kinematic features and the HPSQ-C	Kinematic	N.A.

## Data Availability

Data sharing not applicable.

## References

[1] Cutler L., Graham S. (2008). Primary grade writing instruction: A national survey. J. Educ. Psychol..

[2] McMaster E., Roberts T. (2016). Handwriting in 2015: A main occupation for primary school–aged children in the classroom?. J. Occup. Ther. Schools Early Interv..

[3] Jones D., Christensen C.A. (1999). Relationship between automaticity in handwriting and students’ ability to generate written text. J. Educ. Psychol..

[4] Danna J., Longcamp M., Nalborczyk L., Velay J.-L., Commengé C., Jover M. (2022). Interaction between orthographic and graphomotor constraints in learning to write. Learn. Instruct..

[5] Pinto G., Incognito O. (2022). The relationship between emergent drawing, emergent writing, and visual-motor intergraion in preschool children. Infant Child Dev..

[6] Bonoti F., Vlachos F., Metallidou P. (2005). Writing and drawing performance of school age children: Is there any relationship?. School Psychol. Intl..

[7] Palmis S., Danna J., Velay J.-L., Longcamp M. (2017). Motor control of handwriting in the developing brain: A review. Cogn. Neuropsychol..

[8] Chung P.J., Patel D.R., Nizami I. (2020). Disorder of written expression and dysgraphia: Definition, diagnosis, and management. Transl. Pediatr..

[9] Kalenjuk E., Laletas S., Subban P., Wilson S. (2022). A scoping review to map research on children with dysgraphia, their carers, and educators. Austral. J. Learn. Difficult..

[10] Aiswarya G.S., Ponniah R.J. The modularity of dysgraphia. J. Psycholinguist. Res..

[11] Hamstra-Bletz L., Blöte A.W. (1993). A longitudinal study on dysgraphic handwriting in primary school. J. Learn. Disab..

[12] American Psychiatric Association (2013). Diagnostic and Statistical Manual of Mental Disorders (DSM-5®).

[13] Adi-Japha E., Landau Y.E., Frenkel L., Teicher M., Gross-Tsur V., Shalev R.S. (2007). ADHD and dysgraphia: Underlying mechanisms. Cortex.

[14] Barnett A.L., Prunty M. (2020). Handwriting Difficulties in Developmental Coordination Disorder (DCD). Curr. Dev. Disord. Rep..

[15] Biotteau M., Danna J., Baudou E., Puyjarinet F., Velay J.-L., Albaret J.-M., Chaix Y. (2019). Developmental coordination disorder and dysgraphia: Signs and symptoms, diagnosis, and rehabilitation. Neuropsy. Dis. Treat..

[16] Capodieci A., Lachina S., Cornoldi C. (2018). Handwriting difficulties in children with attention deficit hyperactivity disorder (ADHD). Res. Dev. Disab..

[17] Cohen R., Cohen-Kroitoru B., Halevy A., Aharoni S., Aizenberg I., Shuper A. (2019). Handwriting in children with Attention Deficient Hyperactive Disorder: Role of graphology. BMC Pediatr..

[18] Di Brina C., Caravale B., Mirante N. (2021). Handwriting in children with developmental coordination disorder: Is legibility the only indicator of a poor performance?. Occup. Ther. Health Care.

[19] Berninger V.W., May M.O. (2011). Evidence-based diagnosis and treatment for specific learning disabilities involving impairments in written and/or oral language. J. Learn. Disab..

[20] Afonso O., Suárez-Coalla P., Cuetos F. (2020). Writing impairments in Spanish children with developmental dyslexia. J. Learn. Disab..

[21] Alamargot D., Morin M.-F., Simard-Dupuis E. (2020). Handwriting delay in dyslexia: Children at the end of primary school still make numerous short pauses when producing letters. J. Learn. Disab..

[22] Huau A., Velay J.-L., Jover M. (2015). Graphomotor skills in children with developmental coordination disorder (DCD): Handwriting and learning a new letter. Hum. Mov. Sci..

[23] Johnson B.P., Papadopoulos N., Fielding J., Tonge B., Phillips J.G., Rinehart N.J. (2013). Aquantitative comparison of handwriting in children with high-functioning autism andattention deficit hyperactivity disorder. Res. Autism Spectr. Dis..

[24] Jolly C., Jover M., Danna J. Dysgraphia differs between children with developmental coordination disorder and/or reading disorder. J. Learn. Disab..

[25] Sandler A.D., Watson T.E., Footo M., Levine M.D., Coleman W.L., Hooper S.R. (1992). Neurodevelopmental study of writing disorders in middle childhood. J. Dev. Behav. Pediatr..

[26] Sumner E., Connelly V., Barnett A.L. (2013). Children with dyslexia are slow writers because they pause more often and not because they are slow at handwriting execution. Read. Writ..

[27] Prunty M., Barnett A.L. (2020). Accuracy and consistency of letter formation in children with developmental coordination disorder. J. Learn. Disab..

[28] Richards T.L., Grabowski T.J., Boord P., Yagle K., Askren M., Mestre Z., Robinson P., Welker O., Gulliford D., Nagy W. (2015). Contrasting brain patterns of writing-relatedDTI parameters, fMRI connectivity, and DTI–fMRI connectivity correlations in children with and without dysgraphia or dyslexia. NeuroImage Clin..

[29] Gosse C., Van Reybroeck M. (2020). Do children with dyslexia present a handwriting deficit? Impact of word orthographic and graphic complexity on handwriting and spelling performance. Res. Dev. Disab..

[30] Döhla D., Willmes K., Heim S. (2018). Cognitive Profiles of Developmental Dysgraphia. Front. Psychol..

[31] Berninger V., Abbott R., Cook C.R., Nagy W. (2017). Relationships of attention and executive functions to oral language, reading, and writing skills and systems in middle childhood and early adolescence. J. Learn. Disab..

[32] Graham S., Harris K.R. (2000). The role of self-regulation and transcription skills in writing and writing development. Educ. Psychol..

[33] Nielsen S.K., Kelsch K., Miller K. (2017). Occupational therapy interventions for children with attention deficit hyperactivity disorder: A systematic review. Occup. Ther. Mental Health.

[34] Markham L.R. (1976). Influences of handwriting quality on teacher evaluation of written work. Am. Educ. Res. J..

[35] Engel-Yeger B., Nagauker-Yanuv L., Rosenblum S. (2009). Handwriting performance, selfreports, and perceived self-efficacy among children with dysgraphia. Am. J. Occup. Ther..

[36] Graham S., Fishman E.J., Reid R., Hebert M. (2016). Writing characteristics of students with attention deficit hyperactive disorder: A meta-analysis. Learn. Disab. Res. Pract..

[37] Coradinho H., Melo F., Almeida G., Veiga G., Marmeleira J., Teulings H.-L., Matias A.R. (2023). Relationship between product and process characteristics of handwriting skills of children in the second grade of elementary school. Children.

[38] Rosenblum S., Weiss P.L., Parush S. (2003). Product and process evaluation of handwriting difficulties. Educ. Psychol. Rev..

[39] Asselborn T., Chapatte M., Dillenbourg P. (2020). Extending the spectrum of dysgraphia: A data driven strategy to estimate handwriting quality. Sci. Rep..

[40] Drotár P., Dobeš M. (2020). Dysgraphia detection through machine learning. Sci. Rep..

[41] Guilbert J., Alamargot D., Morin M.F. (2019). Handwriting on a tablet screen: Role of visual and proprioceptive feedback in the control of movement by children and adults. Hum. Mov. Sci..

[42] Rosenblum S., Dror G. (2017). Identifying developmental dysgraphia characteristics utilizing handwriting classification methods. IEEE Trans. Hum. Mach. Syst..

[43] Moetesum M., Diaz M., Masroor U., Siddiqi I., Vessio G. (2022). A survey of visual and procedural handwriting analysis for neuropsychological assessment. Neural Comput. Appl..

[44] Hamstra-Bletz E., de Bie J., den Brinker B.P.L.M. (1987). Beknopte Beoordelingsmethode voor Kinderhandschriften/Concise Evaluation Scale for Children’s Handwriting.

[45] Soppelsa R., Albaret J.-M. (2013). BHK Ado.

[46] Cornoldi C., Ferrara R., Re A.M. (2022). BVSCO-3 Batteria per la Valutazione Clinica della SCRITTURA e della Competenza Ortografica–3 [BVSCO-3, Battery for the Assessment of Writing and Spelling Accuracy].

[47] Phelps J., Stempel L., Speck G. (1985). The Children’s Handwriting Scale: A new diagnostic tool. J. Educ. Res..

[48] Phelps J., Stempel L. (1988). The Children’s Handwriting Evaluation Scale for manuscript writing. Read. Improv..

[49] Barnett A., Henderson S., Scheib B., Schulz J. (2009). Development and standardization of a new handwriting speed test: The Detailed Assessment of Speed of Handwriting. Teach. Learn. Writ..

[50] Scott D.H., Moyes F.A., Henderson S.E. (1985). Diagnosis and Remediation of Handwriting Problems.

[51] Amundson S.J. (1995). Evaluation Tool of Children’s Handwriting.

[52] Pouhet A. (2005). L’évaluation de la vitesse d’écriture manuelle à l’aide d’une dictée de niveau progressif: L’EVEDP. Approches Neuropsychologiques des Apprentissages de l’Enfant.

[53] Erez N., Parush S. (1999). The Hebrew Handwriting Evaluation.

[54] Barnett A.L., Prunty M., Rosenblum S. (2018). Development of the handwriting legibility scale (HLS): A preliminary examination of reliability and validity. Res. Dev. Disab..

[55] Pollock N., Lockhart J., Blowes B., Semple K., Webster M., Farhat L., Jacbson J., Bradley J., Brunetti S. (2009). The McMAster Handwriting Assessment Protocol.

[56] Reisman J.E. (1993). Development and reliability of the research version of the Minnesota Handwriting Test. Phys. Occup. Ther. Pediatr..

[57] Reisman J.E. (1999). Minnesota Handwriting Assessment.

[58] Mutti M., Martin N., Sterling H., Spalding N. (2017). QNST-3R: Quick Neurological Screening Test.

[59] Weil M.J., Cunningham Amundson S.J. (1994). Relationship between visuomotor and handwriting skills of children in kindergarten. Am. J. Occup. Ther..

[60] Larsen S.C., Hammill D.D. (1989). Test of Legible Handwriting: An Ecological Approach to Holistic Assessment.

[61] Milone M. (2007). THS-R: Test of Handwriting Skills. Revised.

[62] Chartrel E., Vinter A. (2008). The impact of spatio-temporal constraints on cursive letter handwriting in children. Learn. Instruct..

[63] Fitzpatrick P., Vander Hart N., Cortesa C. (2013). The influence of instructional variables and task constraints on handwriting performance. J. Educ. Res..

[64] Charles M., Soppelsa R., Albaret J.-M. (2003). BHK—Echelle D’évaluation Rapide de L’écriture chez L’enfant.

[65] Rosenblum S. (2008). Development, reliability, and validity of the Handwriting Proficiency Screening Questionnaire (HPSQ). Am. J. Occup. Ther..

[66] Rosenblum S., Gafni Lachter L. (2015). Handwriting Proficiency Screening Questionnaire for Childrne (HPSQ-C); Development, reliability, and validity. Am. J. Occup. Ther..

[67] Santamaria M., Albaret J.-M. (1996). Troubles graphomoteurs chez les enfants d’intelligence supérieure. Evol. Psychomot..

[68] Di Brina C., Rossini G. (2011). Test BHK-Scala Sintetica per la Valutazione della Scrittura in età Evolutiva.

[69] Loizzo A., Zaccaria V., Caravale B., Di Brina C. (2023). Validation of the concise assessment scale for children’s handwriting (BHK) in an Italian population. Children.

[70] Dimauro G., Bevilacqua V., Colizzi L., Di Pierro D. (2020). TestGraphia, a software system for the early diagnosis of dysgraphia. IEEE Access.

[71] Isa I.S., Rahimi W.N.S., Ramlan S.A., Sulaiman S.N. (2019). Automated detection of dyslexia symptom based on handwriting image for primary school children. Proced. Comp. Sci..

[72] Skunda J., Nerusil B., Polec J. Method for Dysgraphia Disorder Detection using Convolutional Neural Network. Proceedings of the 30th International. Conference in Central Europe on Computer Graphics, Visualization and Computer Vision.

[73] Asselborn T., Gargot T., Kidziński Ł., Johal W., Cohen D., Jolly C., Dillenbourg P. (2018). Automated Human-Level Diagnosis of Dysgraphia Using a Consumer Tablet. Npj Dig. Med..

[74] Dankovičová Z., Hurtuk J., Feciľak P. Evaluation of Digitalized Handwriting for Dysgraphia Detection Using Random Forest Classification Method. Proceedings of the 2019 IEEE 17th International Symposium on Intelligent Systems and Informatics (SISY).

[75] Devillaine L., Lambert R., Boutet J., Aloui S., Brault V., Jolly C., Labyt E. (2021). Analysis of Graphomotor Tests with *Machine learning* Algorithms for an Early and Universal Pre-Diagnosis of Dysgraphia. Sensors.

[76] Deschamps L., Devillaine L., Gaffet C., Lambert R., Aloui S., Boutet J., Brault V., Labyt E., Jolly C. (2021). Development of a pre-diagnosis tool based on *machine learning* Algorithms on the BHK test to improve the diagnosis of dysgraphia. Adv. Artif. Intell. Mach. Learn..

[77] Dui L.G., Lunardini F., Termine C., Matteucci M., Stucchi N.A., Borghese N.A., Ferrante S. (2020). A tablet app for handwriting skill screening at the preliteracy stage: Instrument validation study. JMIR Serious Games.

[78] Dui L.G., Calogero E., Malavolti M., Termine C., Matteucci M., Ferrante S. Digital tools for handwriting proficiency evaluation in children. Proceedings of the 2021 IEEE EMBS International Conference on Biomedical and Health Informatics (BHI).

[79] Dui L.G., Lomurno E., Lunardini F., Termine C., Campi A., Matteucci M., Ferrante S. (2022). Identification and characterization of learning weakness from drawing analysis at the pre-literacy stage. Sci. Rep..

[80] Falk T.H., Tam C., Schellnus H., Chau T. (2011). On the development of a computer-based handwriting assessment tool to objectively quantify handwriting proficiency in children. Comp. Meth. Progr. Biomed..

[81] Galaz Z., Mucha J., Zvoncak V., Mekyska J., Smekal Z., Safarova K., Ondrackova A., Urbanke T., Havigerova J.M., Bednarova J. (2020). Advanced parametrization of graphomotor difficulties in school-aged children. IEEE Access.

[82] Gargot T., Asselborn T., Pellerin H., Zammouri I., Anzalone S.M., Casteran L., Johal W., Dillenbourg P., Cohen D., Jolly C. (2020). Acquisition of handwriting in children with and without dysgraphia: A computational approach. PLoS ONE.

[83] Herstic A.Y., Bansil S., Plotkin M., Zabel T.A., Mostofsky S.H. (2022). Validity of an automated handwriting assessment in occupational therapy settings. J. Occup. Ther. Schools Early Interv..

[84] Kedar S.V. (2021). Identifying Learning Disability Through Digital Handwriting Analysis. Turk. J. Comp. Math. Educ. (TURCOMAT).

[85] Kunhoth J., Al Maadeed S., Saleh M., Akbari Y. (2023). Exploration and analysis of On-Surface and In-Air handwriting attributes to improve dysgraphia disorder diagnosis in children based on *machine learning* methods. Biomed. Sign. Process. Control.

[86] Mekyska J., Faundez-Zanuy M., Mzourek Z., Galaz Z., Smekal Z., Rosenblum S. (2016). Identification and rating of developmental dysgraphia by handwriting analysis. IEEE Trans. Hum. Mach. Syst..

[87] Mekyska J., Galaz Z., Safarova K., Zvoncak V., Mucha J., Smekal Z., Ondrackova A., Urbanek T., Havigerova J.M., Bednarova J. Computerised assessment of graphomotor difficulties in a cohort of school-aged children. Proceedings of the 2019 11th International Congress on Ultra Modern Telecommunications and Control Systems and Workshops (ICUMT).

[88] Rosenblum S., Dvorkin A.Y., Weiss P.L. (2006). Automatic segmentation as a tool for examining the handwriting process of children with dysgraphic and proficient handwriting. Hum. Mov. Sci..

[89] Sihwi S.W., Fikri K., Aziz A. (2019). Dysgraphia identification from handwriting with Support Vector Machine method. J. Physics Conf. Series.

[90] Zvoncak V., Mekyska J., Safarova K., Smekal Z., Brezany P. New approach of dysgraphic handwriting analysis based on the tunable Q-factor wavelet transform. Proceedings of the 42nd International Convention on Information and Communication Technology, Electronics and Microelectronics (MIPRO).

[91] Zvoncak V., Mucha J., Galaz Z., Mekyska J., Safarova K., Faundez-Zanuy M. Fractional order derivatives evaluation in computerized assessment of handwriting difficulties in school-aged children. Proceedings of the 11th International Congress on Ultra Modern Telecommunications and Control Systems and Workshops (ICUMT).

[92] Lopez C., Vaivre-Douret L. (2023). Exploratory investigation of handwriting disorders in school-aged children from first to fifth grade. Children.

[93] Deschamps L., Gaffet C., Aloui S., Boutet J., Brault V., Labyt E. (2019). Methodological issues in the creation of a diagnosis tool for dysgraphia. NPJ Dig. Med..

[94] Lambert R., Boutet J., Labyt E., Jolly C. (2023). Analysis of Eye Movements in Children with Developmental Coordination Disorder During a Handwriting Copy Task. Proceedings of the International Graphonomics Conference.

[95] Lopez C., Cannafarina A., Vaivre-Douret L. (2021). Validity of kinematics measures to assess handwriting development and disorders with a graphomotor task. Eur. Psych..

[96] Bublin M., Werner F., Kerschbaumer A., Korak G., Geyer S., Rettinger L., Schönthaler E., Schmid-Kietreiber M. (2023). Handwriting evaluation using deep-leraning with SensoGrip. Sensors.

[97] Lopez C., Vaivre-Douret L. (2023). Concurrent and predictive validity of a cycloid loops copy task to assess handwriting disorders in children. Children.

[98] Safarova K., Mekyska J., Zvoncak V. (2021). Developmental dysgraphia: A new approach to diagnosis. Int. J. Assess. Eval..

